# Social networks in coworking spaces and individual coworker’s creativity

**DOI:** 10.1007/s11846-021-00445-0

**Published:** 2021-02-08

**Authors:** Alexandra Rese, Lars Görmar, Alena Herbig

**Affiliations:** 1 Chair of Marketing & Innovation, University of Bayreuth, Universitätsstraße 30, 95447 Bayreuth, Germany; 2 Chair of Strategic Management and Organization, University of Bayreuth, Prieserstr. 2, 95440 Bayreuth, Germany

**Keywords:** Coworking spaces, Creative performance, Social networks, Governance mechanisms, Core coworking value orientation, L26, M13, M19, O31, O36

## Abstract

Coworking spaces (CWS) are open creative labs that provide a community-like environment and the necessary surroundings for their users to build and maintain networks with different actors inside and outside the CWS. With a wide variety of knowledge and skills available in trusted surroundings as well as similar value orientations, coworkers enjoy favorable conditions to establish their network-style. However, research has not investigated the benefit of coworkers’ social networks as far as their individual creativity is concerned so far. This paper takes several network characteristics into account: structure in terms of network size and centrality in the CWS, but also trusted and reciprocal relationships, supportiveness, diversity of knowledge exchanged, and the individual openness to core coworking values. Based on the literature on social networks and small group research, we developed a research model. We tested it to get deeper insights into the phenomenon by relying on 113 coworkers in 33 private German coworking spaces. The results show that a central position in the CWS allowing for direct exchange and high individual openness to core coworking values positively affects social involvement and the diversity of knowledge exchanged, and finally, a coworker’s individual creativity. Managerial implications include the vital role of a central position in the CWS for creativity and a somewhat balanced composition of coworkers working alone or in a team.

## Introduction

Recent years have seen a rapid development of coworking spaces (CWS), which primarily offer rentable space and flexible work infrastructure (Spinuzzi 2012). By the year 2018, there were 1.65 million coworkers in 18,700 CWS worldwide, with future growth expected (statista.com 2019a, b). CWS provide infrastructure and dedicated space to facilitate professional and social interaction (Bouncken 2018; Bouncken and Reuschl 2018; Cabral and Van Winden 2016; Gandini 2015). Private providers highlight community and cooperation values and emphasize a relaxed working atmosphere and the possibilities for social exchange and networking (Bouncken et al. 2017; Fuzi et al. 2014; Moriset 2014). Schmidt and Brinks (2017: 291) subsume CWS under the term “open creative labs” that promote an innovative climate. They are supposed to foster creativity due to individuals and teams working in CWS having the opportunity to interact with others, exchange ideas, receive feedback, build partnerships, create trusted relationships, and collaborate with other users (Bouncken et al. 2018). On the part of coworkers, they mention knowledge exchange and learning from others as the main reasons to use CWS (Parrino 2015). While research has related individual creativity to social networks (Perry-Smith and Shalley 2003; Perry-Smith 2006), despite the claims of CWS to enhance creativity, the impact of network characteristics, e.g., network position, has not been researched so far in the coworking context.

Social network development is well established (Araujo and Easton 1996; Hoang and Antoncic 2003; Slotte-Kock and Coviello 2010; Thornton 1999). It is widely accepted that people who need information set up an information network and will “commit time, energy, travel, and sociability to develop their personal networks” (Stewart 1990: 149). Information can be manifold, and in particular, entrepreneurs need information on diverse aspects. This includes feedback on the business idea, task-related help, and assistance for administration-related issues. Coworkers are freelancers, entrepreneurs, and members of start-ups, often with a professional background in IT, creative industries, media, design, or consulting (Bouncken and Reuschl 2018). Zardini et al. (2018: 1031) describe coworkers’ business networks as “breeding ground for entrepreneurial options”. In CWS, these people from diverse backgrounds often work individually but together (Spinuzzi 2012). Bringing these people together reduces the costs mentioned above and networking efforts in terms of time, energy, and travel. As a consequence, CWS can be great places for building and maintaining professional and private networks (Bouncken et al. 2020b).

Despite the increasing importance of coworking and CWS (statista.com 2019a, b), there is currently a lack of research regarding social networks and the effect of network characteristics on (individual) creativity. So far in this context, there are predominantly conceptual and empirical studies investigating knowledge exchange in CWS concentrating on different antecedents, such as geographical proximity, trust or social interaction, and relating them to the process of knowledge exchange, and finally to outcome variables such as individual performance or creativity (Bouncken and Aslam 2019; Bouncken et al. 2018; Bouncken and Reuschl 2018; Parrino 2015; Rese et al. 2020). Going beyond these findings, our study generates an understanding of how different network characteristics such as structure (network size and centrality in the CWS), content (diversity of knowledge exchanged), supportiveness (emotional support, workplace friendship), and shared coworker governance mechanisms (based on trust, reciprocity) influence an individual coworker’s creativity. Besides, we consider the individual openness to coworking as a personality variable, which is reflected in the importance of core coworking values. With this focus, the present study intends to answer the following research questions: (1) How are network structure and content, supportiveness, governance mechanisms, the individual openness to core coworking values, and creativity related? (2) Can a causal chain be applied with input (enabling) variables positively affecting mediating variables, e.g., supportiveness and the diversity of knowledge exchanged, which enhance individual coworker’s creativity?

We collected data in 33 German private CWS in spring 2018, resulting in 113 interviews with coworkers. Besides, a small calibration sample (n = 15) includes answers from two corporate CWS. We applied structural equation modeling (SEM) to test the proposed hypotheses. Our findings show that in the context of CWS and in line with general network research, structural network characteristics, such as centrality in the CWS or network size, had a positive influence on social involvement with coworkers feeling connected and supporting each other, e.g., with diverse knowledge. Despite the constant change of users, diversity of knowledge exchanged, emotional support, trust, and reciprocity as well as individual CWS value orientation displayed high values. This is due to the critical role of values such as community and openness, which CWS try to embody. We can derive several practical implications for the management of CWS by focusing on measures for the support of workplace friendship, the centrality of coworkers in the CWS, and a careful selection process of coworkers regarding their working way, e.g., alone or in a team.

Our findings contribute to network research in entrepreneurship and research on new ways of working. We focus on network effects on processes and creativity as outcomes. We also contribute to research on innovative climate in the workplace and the emerging field of research on coworking and CWS, particularly taking an individual coworker’s professional network into account. The paper has the following structure: first, we shine a light on related research in the coworking context, the theoretical background surrounding entrepreneurial network formation, and present research hypotheses and the research model. Then we explain the research method and data analysis then present the results. The concluding part contains a discussion and implications for further research as well as managerial implications for CWS providers.

## Literature review and research hypotheses

### CWS as innovative workspaces fostering creativity

The idea of CWS experienced a notable trend since the first opening of a CWS in 2005 in San Francisco (Foertsch and Cagnol 2013; statista.com 2019a, b). It was founded as an opposing model to the non-social business centers and provided a workplace and a social area to establish community, freedom, and communication (Dullroy 2012). The idea spread worldwide, with the first CWS in Germany to go by this name opening in 2009 (Foertsch and Cagnol 2013). The professional space comprises the necessary equipment to conduct business activities depending on the specialization of a CWS. The equipment can range from simple desks with Wi-Fi to fully equipped “do-it-yourself” labs (Johns and Gratton 2013). Cafeterias, lounges, and bars constitute the informal social space that drives networking, knowledge exchange, initiation of collaboration, and joint leisure activities, leading to community formation (Bouncken 2018; Gandini 2015; Garrett et al. 2017; Schopfel et al. 2015). Besides, CWS provide their users with special services such as coaching, training, events (start-up) consulting, or access to networks with externals, such as firms, venture capitalists, or business angels (Capdevila 2015; Spinuzzi 2012). Well-established firms—manufacturers such as Bosch, BMW, and Merck or consulting firms such as PwC—have jumped on this institutional trend and set up internal shared work and social spaces as a mean to foster innovation, networking, and the creativity of their employees (Hanney 2017; Tracey et al. 2011).

The idea of CWS demonstrates the manifestations of new ways of working. Generally, new ways of working describe bundles of practices, especially in human resource management, that aim at improving flexibility, autonomy, and freedom for people who are working (Peters et al. 2014; Gerards et al. 2018). For companies, social interaction in this context is a linchpin, especially for intrapreneurial behavior (Gerards et al. 2020). CWS (social) interaction adds value simply by being present and opening talking-restraints of involved parties, eventually increasing entrepreneurial outcomes (Bouncken et al. 2020a, b; Jeske and Ruwe 2019). Overall, Jeske and Ruwe (2019: 174) emphasize that CWS “provide important sources of support, learning and networking opportunities”. Bouncken et al. (2020a: 1465) highlight the closeness to the entrepreneurship field and describe a CWS as “a real space for entrepreneurship”.

So far, concerning networks and networking opportunities, there is a study by Parrino (2015) performing an ego-centric network analysis in two CWS. She analyzes knowledge exchange within the CWS while also taking coworkers’ ties outside the CWS into account. The study shows that besides geographical proximity, implementing an organizational platform is essential to stimulate knowledge exchange, interaction, and collaboration among coworkers. Bouncken and Aslam (2019) confirmed that geographical proximity fosters knowledge sharing processes by coworkers while relying on in-depth qualitative interviews. Knowledge exchange processes and related antecedents, such as trust or community, have been studied by Bouncken and Reuschl (2018) on a conceptual level and Rese et al. (2020) empirically. The latter found a positive relationship between attitudinal and intentional belief to share knowledge and individual creativity. However, network characteristics were not in the research focus.

### Network formation with a focus on CWS

From the perspective of social network analysis, networks are a group of actors related through ties “with some pattern of contacts or interactions between them” based on friendship or business relationships (Newman 2003: 174). These actors’ central aim is to access resources and derive competitive advantage without financial engagement (Slotte-Kock and Coviello 2010). This requires a “structural” involvement of the actors in social interactions based on social contacts and social relations (Brüderl and Preisendörfer 1998).

Regarding the actors, CWS provide office and social spaces for startups, freelancers, and small/entrepreneurial businesses who do not want to lease their own office but want to “interact, share, build, and co-create” (Fuzi 2015: 462). In this sense, CWS and their individual level of diversity fosters co-creation (Görmar et al. 2020). Research has highlighted the role of networks when founding and establishing a new business: the importance of the entrepreneur’s personal networks in the founding process (Birley 1985) and for the later business success (survival, growth) (Brüderl and Preisendörfer 1998). Accordingly, Hoang and Antoncic (2003: 166) identified three key elements in their literature review on how entrepreneurs use, build, and coordinate personal networks and their effect on business outcomes: network content, governance mechanisms, and network structure. Although all elements are closely linked (Hoang and Yi 2015), network-oriented research in entrepreneurship usually focuses on either the structure or the relationships (Slotte-Kock and Coviello 2010).

Regarding the analysis level, this study focuses on interpersonal relationships and not on the intra- or inter-organizational level because individuals or small entrepreneurial or company teams predominantly use CWS (Phelps et al. 2012).

The process of an entrepreneur forming the network starts with individual relationships between two actors, so-called dyads. Their use follows the exploration and selection of dyadic bonds. In the early stage, a broad, diversified social network to receive information and advice to identify entrepreneurial opportunities is essential (Butler and Hansen 1991). Slotte-Kock and Coviello (2010: 35) emphasize that “ties are differentiated not only by intensity but also the content of the relationship”. Strong ties are considered to be useful in terms of trust in information (Jack 2005). For entrepreneurs, relational embeddedness is essential; e.g., they should continue to actively operate in their network and maintain their relationships (Slotte-Kock and Coviello 2010). At a later stage, entrepreneurs can also reactivate and use dormant relationships (Jack 2005).

Various tangible and intangible resources such as capital, business information, advice, emotional support, reputational or signaling content can be exchanged and accessed through interpersonal relationships (Hoang and Antoncic 2003). However, social interactions must not be based on the fulfillment of goals but result from chance (Perry-Smith and Mannucci 2017). CWS reflect places for random encounters, where coworkers often had no contact with each other before joining the coworking space (Brinks 2012; Merkel 2015).

Within a network, an actor can share relationships with different actors, but also different types of relationships with one or more actors. Burt (2000) distinguished broadly between personal and work relationships, which in turn can be positive, but also negative. So-called multiplex ties can involve information, friendship, material, and workflow or competencies (Hoang and Antoncic 2003; Human and Provan 1997; Katz et al. 2004). In the context of CWS it is often referred to the concept of sociomateriality (Aslam and Görmar 2018; Bouncken et al. 2021). Human and Provan (1997) showed that small and medium firms participating in a network had more multiplex relationships than market firms. Hoang and Antoncic (2003) emphasize entrepreneurship research on exchanging intangible resources, e.g., sharing information, collaborative problem solving, and emotional support. CWS, in particular, foster this type of exchange due to the physical proximity of diverse users and playing an active role in initiating and coordinating social interactions (Bilandzic and Foth 2013; Bouncken and Reuschl 2018; Brinks 2012). As a business exchange platform offering a creative and cooperative working atmosphere, coworking places can support their members’ informal interconnection and networking (Bouncken et al. 2020b; Brinks 2012). The “culture of sharing” refers to intangible resources such as knowledge, ideas, and competencies (Brinks 2012). In particular, the core coworking values reflect supportiveness and cooperation (Merkel 2015; Moriset 2014). However, coworkers themselves decide the extent to which they engage in networks and exchange, often working “alone together” (Spinuzzi 2012: 433). Therefore, differences can be expected in coworkers’ individual openness to coworking values.

### Hypotheses development

Overall, in this study, the describing elements are (1) network content where we focus on the diversity of knowledge exchanged, (2) network structure in terms of network size and centrality in the CWS, (3) network governance in terms of trust and reciprocity, and (4) supportiveness based on workplace friendship and emotional support (Hoang and Antoncic 2003). Besides, (5) we included the individual openness to core coworking values (individual CWS value orientation) (Baer 2010). We analyzed these elements on an individual level (Phelps et al. 2012) and modeled the relationships between them as well as their effect on (6) creativity. Concerning the phase of idea generation, this study concentrates on the early idea initiation and elaboration phases (Kijkuit and van den Ende 2010; Perry-Smith und Mannucci 2017).

We transfer conceptual ideas from team research and more precisely rely on the input-process-output model (McGrath 1964; Gladstein 1984; Stock 2014) and resource dependency theory (Pfeffer 1982; Pfeffer and Salancik 1978). We propose a causal chain that starts with enabling factors such as measures of the network structure and individual CWS value orientation but also trust. Individual creativity is mediated by group process variables, with one of the concepts being supportiveness (Gladstein 1984). The network content variable is also conceptualized as a mediator since sharing information and knowledge results in more or less diverse knowledge. These are then related to creativity.

Regarding input factors, a group’s size has been established as a structural characteristic in team research (Gladstein 1984). Network size and centrality of the actor are measures commonly used in network research to describe personal networks (Hoang and Antoncic 2003). They are integrated as input variables (enablers) into the causal chain in the context of coworking. Network structure refers to “the pattern of direct and indirect ties between actors” (Hoang and Antoncic (2003: 170), with network size corresponding to the number of direct ties and centrality to the importance of a node. Both variables serve as a transfer mechanism for knowledge and resources (Fayolle et al. 2011; Ibarra 1993). As another input variable, Gladstein (1984) and Stock (2014) mention the openness regarding specific work norms. According to Stock (2014), the ability and willingness to exchange ideas are higher for open coworkers. In a CWS, like-minded people similarly work alongside each other pursuing entrepreneurial goals (Moriset 2014). The providers established sharing and following mutual norms and values, e.g., core CWS values (Merkel 2015). Due to these shared values and ideas in a CWS, there is a high level of supportiveness in terms of reciprocity and solidarity (Brinks 2012; Fuzi et al. 2014; Merkel 2015; Rus and Orel 2015).

These insights point to the mediating variables. We concentrate on the one hand on network governance mechanisms for coordinating and regulating help (Hoang and Antoncic 2003; Hoang and Yi 2015). Within CWS, coworker-governed networks are prevalent and somewhat informal, with coworkers themselves governing their relationships inside and outside the CWS corresponding to small-firm networks (Balestrin et al. 2008; Parrino 2015). At the same time, coworkers share governance by “interacting on a relatively equal basis in the process of governance” (Provan and Kenis 2008: 234). Essential mechanisms are trust (Larson 1992; Tsai and Ghoshal 1998) and reciprocity (Hoppner et al. 2015). While there is a close relationship between trust and reciprocity (Chaudhuri et al. 2002), the meta-analysis of Bellucci et al. (2017: 1243) analyzing neuroimaging studies found evidence that both concepts “rest on different cognitive processes” because they stimulate different brain regions. Behavioral trust or trust in reciprocity is one facet of trust (Bellucci et al. 2017). Trust is conceptualized here for initial situations in CWS as a precursor of reciprocity. According to Pillutla et al. (2003: 448) “reciprocation of an initially trusting act can instigate a beneficial cycle of increasing trust and reciprocation”.

On the other hand, we concentrate on facets of supportiveness (Gladstein 1984). The coworking context mentions workplace friendship and emotional support (Spinuzzi et al. 2019: 131). Merkel (2015) points to names of coworking spaces such as “Camaraderie” reflecting a work style based on friendship and providing emotional support: coworkers liking each other, sharing and discussing work-related and personal issues, socializing at lunch or after work, and giving each other a helping hand (Simonelli et al. 2018; Toomer et al. 2018). Reciprocity is subsumed together with the two facets of supportiveness (workplace friendship, emotional support) under the term “social involvement”. Finally, we conceptualize the diversity of knowledge exchanged between coworkers as a mediator because it can enhance creativity in the context of networking activities (Wang et al. 2018). We then relate the mediating variables to creativity. Individual creativity has been defined, for example, by Perry-Smith and Shalley (2003: 90) “as an approach to work that leads to the generation of novel and appropriate ideas, processes, or solutions”.

#### Precursors of the diversity of knowledge exchanged and social involvement

##### Network size

Anderson (2008: 53) defines network size as “the number of contacts an actor has” The size of the network determines the extent of access to resources, capabilities, and information, particularly to diverse information (Anderson 2008; Haleblian and Finkelstein 1993; Kijkuit and van den Ende 2010). Besides information and ideas, the number of potential solution strategies and critical judgments as well as the range of perspectives concerning problems, increases (Haleblian and Finkelstein 1993). Therefore, uncertainties and ambiguities can be reduced (Kijkuit and van den Ende 2010). There is some research in favor of a curvilinear relationship of network size and information sharing due to information overload, less involvement, and distraction (Mehra et al. 2001; Zhou et al. 2009). However, these studies investigated employees of large companies. With CWS being much smaller with, on average 68 members in Germany (Deskmag 2018) and the self-employed working in the main by themselves, we expect, corresponding to Kijkuit and van den Ende (2010), that larger spaces are beneficial for providing heterogeneous information and diverse perspectives. A large personal network with a relatively low density and weak ties fosters the exchange of knowledge with diverse information sources (Burt 1992). Anderson (2008) worked out that managers with a high need for cognition and a large network spend more time searching for and finding more information. Regarding social involvement, we expect no effects since there are time and resource restrictions for coworkers who can only directly interact with a limited number of other coworkers regardless of new communication opportunities through social networks (Mayhew and Levinger 1976; Yau et al. 2018). This leads to our first hypothesis:

###### **H1**

The larger the network of a coworker, the higher is the diversity of knowledge exchanged.

##### Centrality in the CWS

The centrality of actors is another measure to evaluate their access to information and resources (Hoang and Antoncic 2003; Rowley 1997). Centrality is defined as the location or position of an individual actor “in the network relative to others” (Rowley 1997: 898). It refers to an actor’s direct ties, which can be used for fast communication within the network. Due to a central position within a network, an individual actor has the opportunity to communicate more frequently and to receive more detailed, accurate, relevant, and diverse information from others faster (Phelps et al. 2012; Tang 2016; Tsai and Ghoshal 1998). Perry-Smith (2006: 88) proposes that individuals with a central network position “may be less judgmental and more open-minded in considering and processing different approaches or ways of thinking”. Since the individual actor can provide other coworkers with diverse information or other resources, this benefit can be used when cooperating with others in terms of shared efforts and resources (Burt 1992; Wincent et al. 2010). Due to a central position, the coworker has more ties, alternatives, and better access to others for emotional support and workplace friendship (Lee and Kim 2011). Since hierarchies play no role in CWS (Bouncken and Reuschl 2018), voluntarily provided emotional support and workplace friendship can evolve (Mao 2006). CWS support the development of direct ties of their members through parties and events or educational programs that are also open to non-members (Merkel 2015). These direct ties can function as indirect connections to other people in the future. Therefore, we propose:

###### **H2**

The more central a coworker is positioned in her/his network inside and outside the coworking space, the higher is the (a) diversity of knowledge exchanged, (b) reciprocity with other coworkers, (c) emotional support, and d) workplace friendship.

##### Trust

Trust is the basis for long-lasting, stable relationships (Hoang and Antoncic 2003; Larson 1992). It needs an interaction-based, long-lasting process to build them (Chow and Chan 2008; Tsai and Ghoshal 1998). Considering each other as trustworthy means, both parties believe that the partner will fulfill all assigned tasks comprehensively and on time (Barney and Hansen 1994; Hoang and Yi 2015; Pruitt 1981). This includes trust in the corresponding person as well as their skills (Larson 1992). Furthermore, trust contains the expectation that the other party does not act opportunistically but rather honestly, e.g., concerning knowledge and for the good of both dyads (Bradach and Eccles 1989; Hsu and Chang 2014; Larson 1992).

A basis of trust improves social interactions and eases the access to resources (Chow and Chan 2008; Newbert and Tornikoski 2012). The higher the trust level, the keener partners actively engage in knowledge exchange (Chow and Chan 2008; Hashim and Tan 2015; Hsu and Chang 2014; Lin 2007). Therefore, trust can be described as a precursor to collaboration and reciprocity (Newell et al. 2007; Zur et al. 2012).

People considered trustworthy are more likely to get help and support from others than those who are not regarded as reliable (Tsai and Ghoshal 1998). Especially in risky and insecure situations, trust combined with emotional support becomes a crucial factor (Hsu and Chang 2014; Larson 1992). When reaching a certain level of trust, actors are willing to join work-related cooperation and hold back from competitive behavior (Larson 1992). People with like-minded work-values and job attitudes as propagated and lived in CWS tend to engage faster in trusting relationships and friendships (Barber 1983; Dotan 2007; Gandini 2015). However, due to the composition of the CWS of like-minded people, we expect no effect of trust on the diversity of knowledge exchanged (Watson et al. 1993). The same holds for the relationship with workplace friendship. While research often conceptualizes trust as a precursor of friendship increasing intimacy in communication (Sias and Cahill 1998), Volker (2019) argues that friendship can also be related to mistrust or established despite trust being somewhat selective. Based on this, we hypothesize:

###### **H3**

The more trustworthy other coworkers are considered, e.g., regarding information exchange, the higher is a) reciprocity with other coworkers, and b) emotional support.

##### Individual CWS value orientation

CWS values are a set of shared visions, norms, and values (Chiu et al. 2006; Tsai and Ghoshal 1998). Their purpose includes providing “shared representations, interpretations, and systems of meaning among parties” (Nahapiet and Ghoshal 1998: 244). They are proposed “to encourage the development of trusting relationships” (Tsai and Ghoshal 1998: 466) and enhance the formation of partnerships. For example, Tsai and Ghoshal (1998: 467) describe visions as a “bonding mechanism” and manifestation of “the collective goals and aspirations of the members of an organisation”. In general, people can expect that these values are valid for all members reducing misunderstandings and conflicts and increasing the frequency and value of knowledge sharing (Chiu et al. 2006; Tsai and Ghoshal 1998).

We regard individual CWS value orientation here as a coworker’s personality characteristic, taking the core value “openness”, for example, to experience, into account (Anderson 2008; Baer 2010; Zhou et al. 2009). Scholars proposed and showed that the need for cognition is a precursor of openness to experience, e.g., “that persons high in need for cognition are intrinsically motivated intellectually, tend to exhibit curiosity, and are tolerant of different ideas” (Sadowski and Cogburn 1997). In particular, we expect the two core values, “cooperation” and “community” to make sharing knowledge more likely and support social involvement in terms of reciprocity, workplace friendship, and emotional support. On the other hand, due to coworkers’ homogenous values, there is no effect on the diversity of knowledge exchanged (Watson et al. 1993). We, therefore, propose the following hypothesis.

###### **H4**

The higher the coworker’s individual openness to coworking values (individual CWS value orientation), the higher is the (a) reciprocity with other coworkers, (b) emotional support, and (c) workplace friendship.

#### Effects of the diversity of knowledge exchanged and social involvement on creativity

##### Diversity of knowledge exchanged (network content)

Personal knowledge is based on an individual’s information and experience “related to facts, procedures, concepts, interpretations, ideas, observations, and judgments” (Yu et al. 2010: 32), and in turn is regarded as one of creativity’s critical drivers (Tang and Ye 2015). Entrepreneurs are interested in suggestions, hints, and ideas for new business opportunities as well as “business information, advice, and problem solving” (Hoang and Antoncic 2003: 169). Research has highlighted the role of individuals’ multiple social and work ties to access a range of diverse work-related knowledge strengthening their creative cognition (Anderson 2008; Baer 2010; Shalley and Perry-Smith 2008; Tang and Ye 2015). Actors within a network can be both the sender and recipient of information, feedback, know-how, or tangible artifacts (Cummings 2004; Phelps et al. 2012). Creativity is also enhanced due to “access and exposure to very different thought worlds”, challenging perspectives, and providing new approaches (Baer 2010: 592). Research has shown that actors strategically include contacts in their personal networks that “they perceive to have more expertise and material resources” (Bridwell-Mitchell and Lant 2014: 401). Besides, knowledge exchange provides an excellent opportunity to expand the personal network with new contacts (Brüderl and Preisendörfer 1998). We, therefore, propose the following hypothesis:

###### **H5**

The higher the diversity of the knowledge exchanged, the higher is an individual coworker’s creativity.

##### Reciprocity

Reciprocity is based on mutuality and establishes the foundations for setting up, maintaining, and using relationships (Hoppner et al. 2015). The literature describes reciprocity as the mutual exchange of favors, with favor from one dyad leading to favor from the other dyad at a later time but for an equal value (Albinsson and Yasanthi Perera 2012; Hoppner et al. 2015; Larson 1992). Gouldner (1960) specifies the factors, naming the dimensions of (1) equality of value which can be expected regarding coworkers and (2) time. Reciprocity implies that people expect favor with equal or comparable value within a reasonable time frame in return when giving favor. Reciprocity acts as an expectation management mechanism, easing, guiding, and stabilizing interactions in a network (Gouldner 1960; Hoppner et al. 2015). As long as a person fulfills the expectations regarding the exchange and the time perspective, it is considered trustworthy (Larson 1992). Consequently, reciprocity enhances the exchange of knowledge (Chang and Chuang 2011; Chiu et al. 2006), and thus creativity. Therefore, we assume:

###### **H6**

The higher the reciprocity between coworkers, the higher is an individual coworker’s creativity.

##### Emotional support

Besides access to information and advice for problem-solving, relational ties provide emotional support (Anderson et al. 2005; Hoang and Antoncic 2003) such as the provision of acceptance, encouragement, affection, empathy, love, appreciation, trust, or caring (Langford et al. 1997; Slevin et al. 1996). Close ties such as those to the family are of particular importance for founders in the start-up phase, providing security and stability while facing risks and uncertainties. Problems and difficulties can be openly addressed and discussed (Brüderl and Preisendörfer 1998). Research has proposed and shown that close and supportive working and non-work relationships provide a positive effect and energy for creative cognition (Madjar et al. 2002; Shalley et al. 2004; Sosa 2011; De Stobbeleir et al. 2011). Closely related to supportive behavior are positive moods such as optimism, confidence, or enthusiasm, which facilitate creativity due to integrative and inductive thinking (George 2000; Isen 1999; Madjar et al. 2002). CWS aim to provide a supportive atmosphere fostering emotional support, friendship, encouragement, and synergies in businesses (Spinuzzi 2012; Gerdenitsch et al. 2016). Since sharing ideas, methods, or techniques and getting feedback, is an essential asset for coworkers (Spinuzzi 2012), opinions, suggestions, and contributions of coworkers should be valued and discussed respectfully. Overall, the following hypothesis arises:

###### **H7**

The higher the emotional support by other coworkers, the higher is an individual coworker’s creativity.

##### Workplace friendship

Emotional support is also closely related to workplace friendship. Pillemer and Rothbard (2018: 3) define the latter as “as a nonromantic, voluntary, and informal relationship between current coworkers that is characterized by communal norms and socioemotional goals”. Several authors emphasize the need to belong to, for example, a work team and be related to others at the workplace (see Pillemer and Rothbard 2018). When actors support each other, this enhances interpersonal affiliation, intimacy, easy conflict resolution, and a sense of family and belonging (Im et al. 2013; Yu et al. 2010), e.g., to a coworking space. Workplace friendship facilitates cooperative behavior and positively affects creative performance (Pillemer and Rothbard 2018). This is due to enhanced communication and interaction, which should be direct and frequent, as well as citizenship and socialization (Im et al. 2013). The resulting close personal relationships can include spending free time together (Burt 2000). If all involved parties are in close contact and equally trust each other, a community can evolve (Kozinets 1999; Sosa 2011; Tang and Ding 2014). A designated space for social interactions offers more direct exchange opportunities and consequently improves the community-building process (Kozinets 1999; Tang and Ding 2014). This space can be found in CWS.

Concerning creativity, one stream of research emphasizes the positive effects of social involvement. The closeness of the involved parties results in emotional support, which reduces uncertainties (Perry-Smith and Mannucci 2017). The closer the parties are, the more open, more intimate, and more honest are the discussions, and the more prone the parties involved are towards giving and receiving feedback, advice, or other forms of help (Gruenfeld et al. 1996; Perry-Smith and Mannucci 2017). Eventually, this improves the exchange of knowledge and resources, problem-solving (Yu et al. 2010), and entrepreneurs’ business ideas. Coworkers with rewarding personal relationships are satisfied by working and accomplishing tasks together (Nielsen et al. 2000). However, there is also the risk of actors becoming too similar and converging in thinking with a negative effect on creativity (Im et al. 2013; Yu et al. 2010). However, we expect this effect not to be prevailing in CWS since Spinuzzi (2012: 433) found that coworkers often work “in the peripheries of each other’s activities—working alone together”. Therefore, we developed the following hypothesis:

###### **H8**

The higher workplace friendship among coworkers, the higher is an individual coworker’s creativity.

**Fig. 1:**
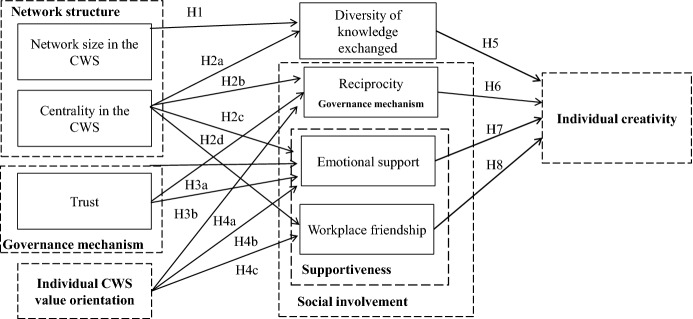
Research model

Figure 1 summarizes the research model tested via the proposed hypotheses.

## The empirical study

### Data collection and questionnaire design

A search was made for existing CWS in Germany, resulting in 307 CWS in 90 cities in the first step. Based on these contact details in terms of location in a federal German state and the town’s size, a sample selection was made while attempting to achieve a well-balanced subsample in terms of German geography and the pattern of population distribution. In particular, we selected more CWS in the south and east of Germany for the sample (see Table 1). As a consequence, the number of CWS in metropolitan cities increased from 63.5 to 87.3%. However, this is in line with another study reviewing the situation of CWS in Germany and showing that almost 90% of the CWS are in large metropolitan cities (Pink 2018).

**Table 1:** Population and sample selection

	German population distribution (31 December 2017) (%)	CWS population (n = 307) (%)	CWS sample (n = 63) (%)	Chi square (*p*, two-sided)
East Germany (without Berlin)	16	4.6	9.5	4.487 (0.045)
West Germany (with Berlin)	84	95.4	90.5	
North Germany	49.8	56.0	36.5	12.257 (0.001)
South Germany	50.2	44.0	63.5	
Metropolitan city	16.9	63.5	87.3	19.350 (0.000)
Non-metropolitan city	83.1	36.5	12.7	

We selected CWS that meet the five core values of coworking. In this understanding, large CWS like WeWork were considered as workplaces and excluded. They contradict the core value “openness” (too high number of individual offices as well as team offices; no exchange with “travelers”) and “community” (no/hardly any community events and no understanding as “WeWork” coworkers). For the data collection, we contacted 63 CWS in 10 major German cities. Slightly more than half (n = 33) participated in the data collection. Data was collected both in-person on-site as well as via an online questionnaire between March 1 and May 8, 2018. Two online responses of coworkers included no information about the CWS in which they currently worked. In personal interviews, 128 questionnaires were collected, of which 103 (80.47%) were complete and usable (offline sample). The online survey yielded ten usable questionnaires (26.32%) out of 38 answers (online sample). Overall, we generated a data set of 166 responses with 113 usable questionnaires. Each CWS contributed between 1 and 8 questionnaires, with an average of 3.36 questionnaires per CWS. To test for possible response biases, we compared the items of the items scales for the online and offline sample with a Mann–Whitney *U*-test. We found no significant differences at the 0.05 level between the two groups. Besides, we were able to collect data at two corporate CWS. We used the results of this small sample of 15 respondents for the research outlook.

We developed the questionnaire based on the literature on topics relating to coworking and CWS, as well as networks and creativity. For our model, we included several multi-item scales, which had to be assessed on a seven-point Likert scale (1 = “strongly disagree” to 7 = “strongly agree”). This involved the following constructs: supportiveness (emotional support, workplace friendship), governance mechanisms (trust, reciprocity), network structure (network size, centrality in the CWS), network content (diversity of knowledge exchanged), individual openness to coworking values and the dependent variable individual coworker’s creativity. Additionally, we integrated measures for the network size, e.g., no. of work ties, no. of friendship ties, no. of first contact ties, and no. of outside ties. For a detailed overview of the items and constructs, see “Appendix A”. An English version of the questionnaire also addressed international coworkers in German CWS.

### Measure validation

We tested the reliability, validity, and uni-dimensionality of each construct’s item scales, relying on SPSS 25 and SmartPLS 3 when calculating exploratory and confirmatory factor analysis (Gerbing and Hamilton 1996). For most constructs, items had to be excluded, e.g., due to their low explanatory power or a VIF above 5 (see Table 2 and “Appendix A”). This also holds for the construct “diversity”, which was not included in the model but used for descriptive purposes (see the item list in “Appendix A”). Because we removed the item referring to the ties outside the network, we needed to restrict the construct to the internal network size and termed “Network size in the CWS”. Concerning individual CWS value orientation, all items were retained to include the facets of CWS values despite an AVE below 0.5 since all items loaded on one factor. To test for common method bias, seven of the model constructs were connected as independent factors to the eighth construct acting as a dependent factor. All eight constructs were used once as a dependent factor. In all cases, the VIFs of the model constructs were lower than the proposed threshold value 3.3 (Kock 2015).

**Table 2:** Summary statistics of measurement scales

Construct	(Original) Number of items	Mean (SD)	Cronbach’s Alpha	Variance explained	CR	AVE
Mutual support						
Emotional support	(6) 4	5.70 (0.97)	0.822	69.464	0.884	0.657
Workplace friendship	(7) 5	3.95 (1.44)	0.892	69.869	0.920	0.698
Governance mechanisms						
Trust	(5) 3	5.32 (1.19)	0.857	77.722	0.913	0,777
Reciprocity	(5) 4	5.79 (1.07)	0.826	65.811	0.885	0.657
Network structure						
Centrality in the CWS	(4) 4	4.14 (1.44)	0.851	69.179	0.899	0.690
Network size in the CWS*	(4) 3	3.29 (3.74)	0.809	72.480	0.887	0.724
Individual CWS value orientation	(5) 5	5.61 (0.90)	0.734	48.957	0.824	0.485
Individual creativity	(8) 7	4.64 (1.34)	0.928	70.058	0.942	0.700

N = 113

Scale: 1 = ‘strongly disagree’ to 7 = ‘strongly agree’

*AVE* average variance extracted, *CR* composite reliability

*In numbers

Except for the AVE of this construct, the suggested thresholds of Cronbach’s α (> 0.7) (Nunnally 1978), composite reliability (> 0.6) (Bagozzi and Yi 1988), and average variance extracted (AVE) (> 0.5) were met (Fornell and Larcker 1981). We did not find any correlation above the threshold of 0.65, indicating multicollinearity (see Table 3) (Grewal et al. 2004). Besides, the criteria for discriminant validity were met: the square root of AVE of the constructs was higher than the correlation of the constructs (Fornell and Larcker 1981), the HTMT ratio of correlations did not exceed 0.85 (Henseler et al. 2015) (see Table 3) and the value 1 was not included in the bias-corrected 95% confidence intervals relying on bootstrapping with 5,000 samples (see Table 8 in “Appendix B”). The sample size was considered large enough. The minimum-squared method (Kock and Hadaya 2018) points to a value a little higher than 65. Soper’s (2004–2020) sample size calculation tool based on the algorithm of Westland (2010) resulted in a minimum sample size of 100 respondents.

**Table 3:** Discriminant validity (Fornell-Larcker criterion; HTMT criterion)

Fornell-Larcker criterion (HTMT criterion)		1	2	3	4	5	6	7	8	9
1	Individual CWS value orientation	**0.696**								
2	Emotional support	0.463 (0.601)	**0.811**							
3	Individual creativity	0.619 (0.742)	0.542 (0.612)	**0.837**						
4	Centrality in the CWS	0.378 (0.469)	0.406 (0.471)	0.535 (0.594)	**0.831**					
5	Reciprocity	0.506 (0.624)	0.606 (0.735)	0.576 (0.641)	0.491 (0.574)	**0.811**				
6	Network size in the CWS	0.188 (0.267)	0.044 (0.154)	0.163 (0.201)	0.296 (0.363)	0.124 (0.181)	**0.851**			
7	Workplace friendship	0.506 (0.593)	0.521 (0.596)	0.546 (0.593)	0.589 (0.670)	0.476 (0.539)	0.333 (0.390)	**0.835**		
8	Trust	0.347 (0.427)	0.491 (0.585)	0.402 (0.439)	0.395 (0.455)	0.572 (0.683)	0.092 (0.131)	0.230 (0.247)	**0.881**	
9	Diversity of knowledge exchange	0.334 (0.385)	0.412 (0.456)	0.478 (0.491)	0.451 (0.481)	0.375 (0.413)	0.312 (0.342)	0.413 (0.436)	0.312 (0.337)	**1.000**

In bold: square root of average variance extracted estimates

N = 113

We calculated a composite score, including all items belonging to that construct for mean value calculation. The value for reciprocity is highest with 5.79 on a scale from 1 = “strongly disagree” to 7 = “strongly agree”, while the lowest value was for network size in the CWS measured numerically at 3.29.

## Research results

### Descriptive results

Our survey coworkers were, for the main part, male, rather young, and often lived in small households with one (20.7%) or two persons (48.6%). More than a quarter were international users. Concerning the academic and professional level, the results are similar to Bouncken and Reuschl (2018): The educational level is very high with predominantly academics who worked as freelancers or in small companies, and in branches such as IT, consulting, or creative industries. The coworkers used CWS in general for about one and a half years and the current space for a little more than a year. Of the CWS core values, they highlight in particular openness, e.g., “free sharing of ideas, information and people” (Fuzi et al. 2014: 2), and financial and physical accessibility (Moriset, 2014), but to a lesser extent collaboration (see Table 4).

**Table 4:** Coworker characteristics

Properties	Sample
Gender	
Females	31.0%
Males	69.0%
Age	
Mean value (SD)	32.20 (6.762)
Up to 29	36.6%
30 up to 39	52.7%
40 and older	10.7%
Nationality	
German	69.9%
Other	30.1%
Family status single	78.8%
Education	
Ph.D.	6.2%
Diploma, Magister, state exams	15.9%
Master degree	28.3%
Bachelor degree	35.4%
Profession	
Web develop-ment/IT	22.1%
Consulting	15.0%
Marketing	13.3%
Creative industries, design	23.9%
Occupation group	
Freelancer	32.7%
Entrepreneur	19.5%
Enterprise with up to 5 employees	15.0%
Enterprise with 6 up to 99 employees	22.1%
Enterprise with > 100 employees	8.8%
Location of CWS	
Berlin	36.9%
Munich	11.7%
Leipzig, Stuttgart	9.9%
Period of use (in months)	
In general	19.13 (15.390)
This CWS	14.46 (11.268)
Individual CWS value orientation*	
Collaboration (mean, SD)	4.81 (1.618)
Community (mean, SD)	5.82 (1.241)
Sustainability (mean, SD)	5.42 (1.474)
Openness (mean, SD)	5.95 (1.016)
Accessibility (mean, SD)	6.04 (1.117)

*Scale: 1 = ‘strongly disagree’ to 7 = ‘strongly agree’

The respondents work in relatively small CWS with average 25 members compared to the 68 members in the Deskmag (2018) study, including large workplaces. They rate their coworkers to be somewhat diverse in terms of knowledge, skills, educational background, and way of thinking. About half of the coworkers are working alone. The other half is working in teams with about three to four persons for a little more than a year. For those working in a team, the value of “collaboration” was higher (5.12 vs. 4.49, *p* = 0.022) and “accessibility” lower, presumably due to the presence of other team members (5.79 vs. 6.29, *p* = 0.026). Coworkers were most likely to share knowledge and ideas with others, followed by specific expertise and helpful advice to solve problems. Regarding entrepreneurial activities, about half of the coworkers exchanged ideas and suggestions for new business ideas as well as new potential interesting contacts. On average, coworkers gain access to 5.24 types of knowledge through networking in their CWS. Respondents working in a team shared valuable business information (27.9% vs. 21.8%) and solutions for work problems (29.7% vs. 21.8%) more frequently. Regarding their network inside the CWS, respondents had started with having contact with about one coworker. The direct work-related exchange increased in the meantime to about five coworkers, and with about three of them, they are befriended. While team coworkers’ network is more extensive (6 up to 7 persons compared to 3 up to 4 persons), the number is similarly high without the team members. Outside the CWS, respondents have a direct work-related exchange with about 14 persons (Table 5).

**Table 5:** Networking characteristics

Properties	Sample
Size of CWS	
Mean value (SD)	24.79 (20.975)
Minimum/Maximum	2/150
Diversity of coworkers	
Mean value (SD)	5.33 (1.14)
Team	
Working in team	50.2%
Team size	3.54 (4.390)
Duration of collaboration (months)	15.000 (19.484)
Diversity of knowledge exchanged	
Mean value (SD)	5.14 (2.783)
Knowledge and ideas	79.9% (n = 90)
Specific expertise	60.2% (n = 68)
Specific skills	39.8% (n = 45)
Valuable business information	30.1% (n = 34)
Access to services	50.4% (n = 57)
Helpful advice to solve problems	53.1% (n = 60)
Solutions for work problems	31.0% (n = 35)
General practical “hands on” advice and assistance	41.6% (n = 47)
Latest information on current business topics	30.1% (n = 34)
Ideas and suggestions for new business ideas	44.2% (n = 50)
New, potential contacts	49.6% (n = 56)
Direct work related exchange inside the CWS	
Mean value (SD)	5.29 (6.75)
Minimum/Maximum	0/40
With a friendly relationship	2.76 (3.467)
In % of work related exchange	59.86 (39.16)
Contact from the beginning	1.18 (1.767)
In % of work related exchange	39.42 (42.21)
Direct work-related exchange outside the CWS	
Mean value (SD)	14.08 (27.465)
Minimum/Maximum	0/200

### Hypothesis testing

The path coefficients’ significance is established with a *p* value below 0.05 and bias-corrected confidence intervals, excluding the value zero, particularly the case here. The bias-corrected confidence intervals are above zero for all path coefficients with a *p*-value below 0.05, thus reinforcing the significance (Ringle et al. 2018).

Regarding the model, our data confirmed almost all hypotheses except the proposed effect of centrality on emotional support (H2c) and emotional support on creativity (H7). For the precursors of knowledge exchanged and social involvement, in particular, the centrality of the coworkers in the CWS (0.464, *p* = 0.00) and a high individual CWS value orientation (0.331, *p* = 0.00) had a positive effect on workplace friendship (see Table 6). In addition, a central position in the CWS (0.393, *p* = 0.000) was important for the diversity of knowledge exchanged followed by the size of the network in the CWS (0.196, *p* = 0.037). Like individual CWS value orientation, trust influences social involvement in terms of reciprocity (0.382, *p* = 0.005) and emotional support (0.325, *p* = 0.012), thus confirming hypotheses 3a and b. Higher effect sizes f^2^ above 0.15 and described as moderate (Henseler et al. 2009) demonstrated the contribution of centrality in the CWS to workplace friendship (f^2^ = 0.329) and diversity of knowledge exchanged (f^2^ = 0.185). Other moderate effect sizes were found for the contribution of trust to the R^2^ value of reciprocity (f^2^ = 0.223) and the one of individual CWS value orientation to workplace friendship (f^2^ = 0.168).

**Table 6:** Testing the relationships of the research model

Independent variable		Dependent variable	Hypothesis	Path coefficients (Effect size—f^2^)	T statistics (*p*-value)	Bias-corrected confidence interval (95%)
Network size in the CWS	→	Diversity of knowledge exchanged	H1	0.196 (0.046)	2.089 (0.037)*	[0.0002, 0.367]
Centrality in the CWS	→	Diversity of knowledge exchanged	H2a	0.393 (0.185)	5.028 (0.000)***	[0.224, 0.532]
Centrality in the CWS	→	reciprocity	H2b	0.232 (0.080)	3.151 (0.002)**	[0.092, 0.379]
Centrality in the CWS	→	Emotional support	H2c	0.169 (0.035)	1.560 (0.119)	[−0.031, 0.396]
Centrality in the CWS	→	Workplace friendship	H2d	0.464 (0.329)	5.616 (0.000)***	[0.150, 0.489]
Trust	→	Reciprocity	H3a	0.382 (0.223)	2.800 (0.005)**	[0.069, 0.602]
Trust	→	Emotional support	H3b	0.325 (0.132)	2.524 (0.012)*	[0.076, 0.569]
Individual CWS value orientation	→	Reciprocity	H4a	0.286 (0.126)	3.163 (0.002)**	[0.109, 0.450]
Individual CWS value orientation	→	Emotional support	H4b	0.287 (0.104)	2.459 (0.014)*	[0.035, 0.490]
Individual CWS value orientation	→	Workplace friendship	H4c	0.331 (0.168)	3.831 (0.000)***	[0.295, 0.616]
Diversity of knowledge exchanged	→	Individual creativity	H5	0.204 (0.062)	2.060 (0.039)*	[0.012, 0.402]
Reciprocity	→	Individual creativity	H6	0.289 (0.095)	3.254 (0.001)**	[0.104, 0.452]
Emotional support	→	Individual creativity	H7	0.156 (0.026)	1.403 (0.161)	[−0.063, 0.374]
Workplace friendship	→	Individual creativity	H8	0.243 (0.074)	2.734 (0.006)**	[0.057, 0.406]

*Significant with *p* < 0.05, **Significant with *p* < 0.01, ***Significant with *p* < 0.001

Concerning the individual creativity of coworkers, reciprocity (0.289, *p* = 0.001) has the strongest positive effect, followed by workplace friendship (0.243, *p* = 0.006) and diversity of knowledge exchanged (0.204, *p* = 0.039). We found evidence for H5, H6, and H8, but not for emotional support and H7. However, the f^2^ values displayed only smaller effects as they were below 0.15.

Looking at how well-developed the research model is, the R^2^ and R^2^ adjusted values (coefficients of determination) are all above 0.25, which is a weak effect (see Table 7). In particular, individual creativity (0.482), reciprocity (0.477), and workplace friendship (0.440) are close to 0.50, the threshold for being accounted for a moderate effect (Hair et al. 2011).

**Table 7:** Structural model evaluation

	R^2^	R^2^ adj.	Q^2^
Diversity of knowledge exchanged	0.238	0.224	0.202
Reciprocity	0.477	0.463	0.277
Emotional support	0.361	0.344	0.215
Workplace friendship	0.440	0.430	0.296
Individual creativity	0.482	0.462	0.324

## Discussion

In this study, we analyzed the effect of social networks on individual creativity in the context of CWS. Based on the literature, we developed (multi-item) constructs that describe established networking elements such as network content, network structure, network governance, supportiveness, and network structure (Anderson et al. 2005; Brinks 2012; Hoang and Antoncic 2003; Hoppner et al. 2015). With the construct “Individual CWS value orientation”, referring to the individual’s openness to the five core coworking values (Schürmann 2013), a CWS-specific element was added on the individual level (Bouncken et al. 2020b). The constructs were related to each other in a research model based on the input-process-output framework (McGrath 1964; Gladstein 1984; Stock 2014).

The results show that for private CWS, the networks of coworkers within the CWS are rather of the same size regardless of working alone or in a team if subtracting the number of team members. There were some, but not many significant differences, for example, when it comes to the types of knowledge exchanged. When looking at the results of the small sample of corporate CWS, these are somewhat different. Coworkers of corporate CWS predominantly work in a team (93% vs. 50.2%). Not surprisingly, the mean value of the core value “collaboration” is highest with 6.47 compared to 5.12 (private CWS: team worker) and 4.49 (private CWS: working alone). When subtracting the number of team members (7.2), their network within the corporate CWS is smaller (about 2.2), but outside the CWS, their network is more extensive with on average 19.53 contacts compared to 14.08. In contrast, for coworkers in private CWS, their personal network is more diverse when it comes to knowledge and skills, educational background, and way of thinking and action (5.33 vs. 4.18). Concerning entrepreneurial activities, the exchange of ideas and suggestions for new business ideas (33.3% vs. 44.2%) as well as new potential interesting contacts (13.3% vs. 49.6%) are noticeably less frequent.

Our findings demonstrate that networking and related elements positively impact individual creativity for freelancers and entrepreneurs when they work in CWS. Evaluating the research model shows that a central position in the CWS, allowing a direct exchange with other coworkers and a high individual CWS value orientation, significantly influences workplace friendship and reciprocity. For centrality in the CWS, this also holds for the diversity of knowledge exchanged. In addition, trust increases reciprocity and emotional support. The literature on social cohesion is supported in the context of CWS (Burt 2000; Im et al. 2013; Yu et al. 2010). In turn, in particular reciprocity and workplace friendship, but also the diversity of knowledge exchanged, positively affects individual creativity. The first two factors reflect the core ideas of CWS in terms of helping each other and supporting each other with reciprocity, also pointing to the importance of direct exchange. However, emotional support, such as a sense of family and belonging or easy conflict resolution, is less important.

Overall, the incoming factors in our model explain individual creativity, but also reciprocity and workplace friendship quite well. Nevertheless, about 50% of the variance is not explained by these factors. For diversity of knowledge exchanged and emotional support, the unexplained part is even larger. Therefore, the investigation of other factors such as the diversity of network partners or coworkers in the CWS, available resources in the CWS, e.g., training or technical services, or boundary management (Gladstein 1984), as well as other personality factors such as empathy or emotional intelligence would be of interest.

### Theoretical implications

With the research model and the following SEM analysis, we contribute to the literature on coworking and related research, e.g., new ways of working (Gerards et al. 2020), and focus on knowledge exchanged, social interaction, and creative outcomes (Bouncken and Aslam 2019; Garrett et al. 2017; Gerdenitsch et al. 2016; Rese et al. 2020). In particular, we go beyond conceptual (Bouncken and Reuschl 2018) and qualitative analyses (Bouncken and Aslam 2019; Capdevila 2019; Spinuzzi et al. 2019) or literature reviews (Jeske and Ruwe 2019). Relying on network elements from entrepreneurial networking, we can confirm the importance of network content, reciprocity, and supportiveness for individual creativity in the coworking context (Hoang and Antoncic 2003). Individual CWS value orientation proved to be an important precursor of social involvement in terms of reciprocity and supportiveness (Chiu et al. 2006; Tsai and Ghoshal 1998). While the five core values loaded on one factor and uni-dimensionality could be established, the value “collaboration” is a discriminator for those working alone and team workers in the CWS. This is also a finding of Rese et al. (2020) confirming member heterogeneity (Bouncken and Reuschl 2018). In addition, we contribute to research on network structure and similarly to research in other contexts. Wang et al. (2019) found that direct connections (centrality) and the number of connections (network size in the CWS) are important input factors. Taking the proposed function of CWS as open creative labs into account, we contribute to research on innovative climate (Liu et al. 2019). Our results reveal the importance of workplace friendship and reciprocity in the coworking context. For early professional networks, we regarded and confirmed trust as a precursor of reciprocity (Newell et al. 2007; Zur et al. 2012). However, for future research, it has to be considered that trust is also a result of reciprocity developing over time from collaboration with others (Newell et al. 2007).

### Managerial implications

Our findings include important managerial implications for CWS management. First, our results indicate that a central position in the CWS fosters in particular, workplace friendship and diversity of knowledge exchanged. Therefore, the formats of networking events, but also architectural elements, and technical support should ensure that direct exchange with other coworkers is enabled. When CWS support matchmaking and networking with tools (Kopplin 2020), they should take care of as many direct exchange possibilities as possible. The spaces should not be too small, allowing for the opportunity to build networks within the space. Diversity of knowledge exchanged is increased, and there are more possibilities for workplace friendships. In addition, formats fostering workplace friendship should invite coworkers to spend time together and continuously reflect on their effectiveness. CWS managers should be attentive to coworkers in peripheral network positions. They should include coworkers in event planning by asking them what they would like to see/hear/do and how to approach which internal and external parties. Additionally, coworkers should be encouraged to plan their own events that are suitable for the CWS. Since the results confirm the supportive climate in the CWS the necessity for tools or guidelines for conflict management is reduced.

The individual CWS value orientation’s importance calls for a careful selection of coworkers identifying and living the core CWS values. In particular, regarding the diversity of knowledge exchanged, a mix of members working in a team and working alone is advisable. Team coworkers provide more often process-related knowledge, e.g., solutions for work problems (29.7% vs. 21.8%) or general practical “hands-on” advice and assistance, while coworkers working alone offer more specific skills (47.3% vs. 32.8%). A more balanced composition is highly recommended for corporate coworking spaces, with 93% of the members working in a team.

### Limitations and research outlook

Of course, the study is not free of several shortcomings. First, the sample from private CWS is relatively small. The sample from corporate CWS is even smaller but should be of a similar size to allow a comprehensive comparison. In addition, we investigated only German CWS. The results of an international sample would complement the picture (Appel-Meulenbroek et al. 2020). We concentrated our data collection on CWS in metropolitan cities for Germany, where they typically locate. Contrasting the results with an analysis of a sample of coworkers in CWS in small- and medium-sized cities would be interesting. Heterogeneity regarding CWS types and communities could be taken into account (Capdevila 2019; Spinuzzi et al. 2019).

Regarding network size in the CWS, the investigated CWS were on average smaller than German ones in general and might offer limited opportunities for personal network formation. However, there might be a maximum size where the positive properties of large CWS and potential large personal networks are reversed. Therefore, future research should investigate the size of this potential turning point.

Second, we used self-reported dependent and independent variables in this study. The results can be enriched with objective data collected, for example, on essential features of CWS such as price, location, safety, conference rooms, kitchenette, or opening hours. Other researchers can also include other variables, for example, different personality traits such as attitudes and motivational factors to engage with others (Bock et al. 2005).

Third, for each coworker in a CWS, a holistic, ego-centric network could be mapped (Parrino 2015). The evaluation of the nodes in the network regarding important personality traits as well as in-depth structural analyses could enrich the understanding of social interaction and knowledge sharing mechanisms in the CWS (Bouncken and Aslam 2019). Other social network measures, such as network density could be investigated (Marsden 2005). A comparison of the network structure in the CWS with the coworker’s individual network, e.g., to identify structural gaps (Burt 1992, 2004), could also be interesting. Besides the professional network other types of personal networks such as friendship and advice networks could be more in focus (Gibbons 2004) because they enrich the CWS network. Ties outside the CWS had a descriptive character in this study. Still, they could be investigated in more detail together with the strength (duration of contact, frequency of exchange, closeness) and diversity of ties (Perry-Smith 2006).

Fourth, regarding network development, a process orientation can be considered (Hoang and Antoncic 2003). Personal networks are not fixed constructs but are subject to constant change over time, e.g., due to different activation depending on the situational context (Perry-Smith and Mannucci 2017). Looking at the entire network, complexity increases over time as new relationships can arise, enhancing network density and cohesion or structural gaps develop, making the network sparser (Hite and Hesterly 2001; Slotte-Kock and Coviello 2010). Therefore, an investigation at several points in time, for example, in several selected CWS, would offer additional insights. In addition, studying the usability and efficiency of tools to facilitate networking is of interest (Kopplin 2020), particularly against the background of the COVID-19 pandemic.

### Effects of the COVID-19 crisis on future coworking

The latest pandemic left its footprint on the work-life. The corona-virus showed that most of the work does not require a fixed workplace but can be done remotely. An increasing number of companies switched to teleworking, relying on digital technologies, such as Zoom, and alternative workspaces such as CWS seem to become obsolete (Carnevale and Hatak 2020; Sheth 2020). A worldwide survey of CoworkingEurope in the Spring showed that about half of the CWS in Europe (47%) were strongly affected, in particular regarding members staying at home (34%) and events being canceled (20%) (Calders 2020). About 10% of the CWS closed in Germany, and the rest was open with a regular or reduced service (Foertsch 2020b). Not surprisingly, Reuschke and Felstead (2020: 211) raised the question regarding “the future of the collective, open-plan office where desks and equipment are shared and the future viability of promoting co-working spaces where different workers and businesses share the same premises”. The Corona-pandemic challenged social interaction as the basis of success. Working without direct interaction becomes part of everyday life for many people. CWS implemented many distancing measures, such as decreasing the number of desks and seats or closing all meeting rooms (Calders 2020; Foertsch 2020a). While coworking lives from interaction and togetherness, CWS suggested coworkers working from home for 2 weeks or did not allow in guests. However, the pandemic is also seen as a chance for CWS to claim their place in the work environment. CWS have started to adjust their business model temporarily. In particular, they introduced a change in one area: planning to or already offering more online services and motivating coworkers to use digital technologies for working and meeting remotely (Foertsch 2020a, b). Since teleworking has besides benefits also disadvantages (Baruch 2000), experts are calling for “a portfolio of space solutions: owned space, standard leases, flexible leases, flex space, co-working space, and remote work” (Boland et al. 2020: 5). Foertsch (2020b) even describes a bright future for CWS due to increased entrepreneurial activity caused by companies’ lack of work perspectives. CWS can develop a leading role as places where digital business models are created (Bouncken et al. 2020a). Lestari (2020) highlights CWS managers’ role in initiating and supporting collaboration processes among startups in CWS. CWS can offer the service of innovation community building and open innovation process upsetting (Fichter 2009; Rese et al. 2013) to startups and established firms. However, profitability has to be in focus, and operations and the business model of CWS need to be continuously adapted to be competitive in the long-run (Kraus et al. 2020).

## Conclusions

Entrepreneurial networks have been a topic of research interest (Birley 1985; Brüderl and Preisendörfer 1998; Hoang and Antoncic 2003; Hoang and Yi 2015; Slotte-Kock and Coviello 2010). Research has up to know studied entrepreneurs and freelancers in CWS, their professional networks, and the related knowledge exchange to a limited extent (Bouncken and Aslam 2019; Bouncken et al. 2020a, b; Parrino 2015). We developed a research model based on the input-process-output framework (McGrath 1964; Gladstein 1984; Stock 2014) relating network structure and content, supportiveness, governance mechanisms, the individual openness to core coworking values, and individual creativity. A causal chain can be applied, demonstrating the importance of centrality in the network and individual CWS value orientation as input (enabling) variables as well as workplace friendship and reciprocity as mediating variables. While for private CWS we could establish that creativity can be fostered, it remains to be seen whether this concept can be successfully transferred to large companies. Our study gives initial insights that the users of corporate CWS and their professional networks are different.

## Appendices

### Appendix A: Item list (translated from German)

1. Network Content.

*Knowledge exchange* (Anderson et al. 2005; Capdevila 2013; Fuzi et al. 2014; Gerdenitsch et al. 2016; Hoang and Antoncic 2003; Lin 2007; Perry-Smith 2006; Tang and Ding 2014; Tohidinia and Mosakhani 2010).

Because of networking with others in my coworking space, I receive…

1. … access to knowledge and ideas of others.
2. … access to specific expertise. (dropped).
3. … access to specific skills.
4. … valuable business information.
5. … access to services.
6. … helpful advice in solving problems.
7. … solutions for my problems at work.
8. … general practical “hands-on” advice and help.
9. … the latest information about current business issues.
10. … ideas and inspiration for new business ideas.
11. … new, potential customers.
12. Other: ___________________.

(2) Supportiveness.

*Emotional Support* (Brinks 2012; Brüderl and Preisendörfer 1998; Garrett et al. 2017; George 2000; Jehn and Mannix 2001; Lin 2007; Sánchez-Franco and Roldán 2015; Spinuzzi 2012).

At my coworking space…

1. … the mood among coworkers is positive and characterized by optimism.
2. … enthusiasm for new ideas is exciting and motivates me. (dropped).
3. … I can openly speak about all problems and difficulties I have at work. (dropped).
4. … we deal with and discuss suggestions and contributions of members in a respectful way.
5. … coworkers are open-minded and sympathetic to me.
6. … I feel accepted and understood.

*Workplace friendship* (Anderson et al. 2007; Brinks 2012; Capdevila 2013; Garrett et al. 2017; Gerdenitsch et al. 2016; Lin 2007; Nielsen et al. 2000; Sánchez-Franco and Roldán 2015; Spinuzzi 2012).

At my coworking space…

1. … we like to spend time together outside of work.
2. … we stick together and support each other.
3. … we often celebrate together.
4. … we get along well together (dropped).
5. … we all do our own thing (reverse: dropped).
6. … I was able to develop close relationships with the coworkers.
7. … I found personal friends.

(3) Governance Mechanisms.

*Trust* (Jehn and Mannix 2001; Larson 1992; Tang 2016; Tsai and Ghoshal 1998).

At my coworking space…

1. … everyone is honest and sincere in dealing with me in terms of knowledge (dropped).
2. … no one takes advantage of me and my know-how (dropped).
3. … everyone deals constructively and carefully with my information.
4. … the information I receive is totally truthful.
5. … everyone keeps the promises they make to me.

*Reciprocity* (Bock et al. 2005; Chen and Hung 2010; Hoppner et al. 2015; Pai and Tsai 2016)

1. If a coworker helps me, I will try to offer him/her comparable support (dropped).
2. If I receive help in my coworking space, I feel it is only right to help others as well.
3. Members of my coworking space would help me if I need help.
4. I would feel an obligation to help members of the coworking space if they need my support.
5. Solidarity between members plays a very important role in my coworking space.

(4) Network Structure.

*Network size* (Hoang and Antoncic 2003; Hoang and Yi 2015; Tang and Ding 2014; Tang and Ye 2015).

1. Define the number of people within your coworking space you are currently engaged in a direct work-based exchange (Internal).
2. To how many of these coworkers are you closely connected/friendly? (Internal).
3. With how many of these coworkers were you already in contact with from the first moment of using the coworking space? (Internal).
4. Define the number of people outside your coworking space you are currently engaged with for direct work-based exchange (External).

*Centrality in the CWS (*Hoang and Antoncic 2003).

At my coworking space…

1. I directly receive helpful information from each coworker.
2. I can directly ask each coworker for advice.
3. I directly discuss current business issues with each coworker.
4. I quickly receive important news from coworkers.

(5) Individual CWS value orientation (Schürmann 2013).

Rate how important the following core values of a coworking space are to you.

1. Collaboration (dropped).
2. Community (dropped).
3. Sustainability.
4. Openness.
5. Accessibility.

(6) Diversity.

(Baer 2010; Fuzi et al. 2014; Gandini 2015; Mumford and Gustafson 1988; Perry-Smith 2006; Pohler 2012; Spinuzzi 2012; Tang 2016; Tang and Ye 2015).

In my coworking space coworkers differ especially in…

1. … their knowledge and skills.
2. … their educational background.
3. … their way of thinking and course of action.
4. … their views and opinions (world view) (dropped).
5. … their beliefs about what is right or wrong (dropped).

(7) Individual Creativity (Chen et al. 2015; Tang 2016).

Networking with others in my coworking space…

1. … is a good source of new creative ideas.
2. … increases the number of my creative ideas.
3. … increases the originality of my work.
4. … makes me aware of completely new working methods.
5. … helps me to reinterpret my existing ideas.
6. … provides insights into ideas and concepts of others that are useful to my work.
7. … enables me to solve specific problems optimally.
8. … enables me to solve work-related problems creatively.

### Appendix B

See Table 8.

**Table 8:** Discriminant validity (HTMT confidence interval)

	Bias corrected 95% confidence interval
Emotional support -> Individual CWS value orientation	[0.399, 0.827]
Individual creativity -> Individual CWS value orientation	[0.559, 0.880]
Individual creativity -> Emotional support	[0.412, 0.773]
Centrality in the CWS -> Individual CWS value orientation	[0.325, 0.641]
Centrality in the CWS -> Emotional support	[0.291, 0.684]
Centrality in the CWS -> Individual creativity	[0.452, 0.717]
Reciprocity -> Individual CWS value orientation	[0.498, 0.806]
Reciprocity -> Emotional support	[0.548, 0.896]
Reciprocity -> Individual creativity	[0.467, 0.767]
Reciprocity -> Centrality in the CWS	[0.404; 0.716]
Size of network in CWS -> Individual CWS value orientation	[0.189; 0.460]
Network size in the CWS -> Emotional support	[0.110; 0.325]
Network size in the CWS -> Individual creativity	[0.101; 0.359]
Network size in the CWS -> Centrality in the CWS	[0.231; 0.506]
Network size in the CWS -> Reciprocity	[0.098; 0.355]
Workplace friendship -> Individual CWS value orientation	[0.463; 0.757]
Workplace friendship -> Emotional support	[0.422; 0.746]
Workplace friendship -> Individual creativity	[0.410; 0.746]
Workplace friendship -> Centrality in the CWS	[0.512; 0.802]
Workplace friendship -> Reciprocity	[0.392; 0.672]
Workplace friendship -> Network size in the CWS	[0.241, 0.559]
Trust -> Individual CWS value orientation	[0.276, 0.657]
Trust -> Emotional support	[0.360, 0.802]
Trust -> Individual creativity	[0.240, 0.640]
Trust -> Centrality in the CWS	[0.247, 0.650]
Trust -> Reciprocity	[0.376, 0.886]
Trust -> Network size in the CWS	[0.067, 0.276]
Trust -> Workplace friendship	[0.141, 0.458]
Diversity of knowledge exchanged -> Individual CWS value orientation	[0.212, 0.572]
Diversity of knowledge exchanged -> Emotional support	[0.222, 0.648]
Diversity of knowledge exchanged -> Individual creativity	[0.285, 0.657]
Diversity of knowledge exchanged -> Centrality in the CWS	[0.317, 0.628]
Diversity of knowledge exchanged -> Reciprocity	[0.257, 0.541]
Diversity of knowledge exchanged -> Network size in the CWS	[0.129, 0.542]
Diversity of knowledge exchanged -> Workplace friendship	[0.233, 0.614]
Diversity of knowledge exchanged -> Trust	[0.175, 0.489]
